# Supplementary material for: A fault-intrusion-tolerant system and deadline-aware algorithm for scheduling scientific workflow in the cloud
Source: PeerJ Comput Sci. 2021 Nov 2;7:e747. doi: 10.7717/peerj-cs.747 (PMC8576554; doi:10.7717/peerj-cs.747)
Supplement: Supplemental Information 6 [file peerj-cs-07-747-s006.zip › Myproject1.1/docs/org/workflowsim/scheduler/SchedulerInterface.html]

SchedulerInterface


JavaScript is disabled on your browser.


- Overview
- Package
- Class
- Use
- Tree
- Deprecated
- Index
- Help

- Prev Class
- Next Class

- Frames
- No Frames

- All Classes

- Summary:
- Nested |
- Field |
- Constr |
- Method

- Detail:
- Field |
- Constr |
- Method


org.workflowsim.scheduler

## Interface SchedulerInterface

- All Known Implementing Classes:
  :   BaseScheduler, DataAwareScheduler, FCFSScheduler, MaxMinScheduler, MCTScheduler, MinMinScheduler, StaticScheduler

  ---

    

  ```
  public interface SchedulerInterface
  ```

  The Scheduler interface

  Since:
  :   WorkflowSim Toolkit 1.0

- - ### Method Summary

    Methods

    | Modifier and Type | Method and Description |
    | --- | --- |
    | `java.util.List` | `getCloudletList()` Gets the job list. |
    | `java.util.List` | `getScheduledList()` Gets the scheduled jobs. |
    | `java.util.List` | `getVmList()` Gets the vm list. |
    | `void` | `run()` the main function. |
    | `void` | `setCloudletList(java.util.List list)` Sets the job list. |
    | `void` | `setVmList(java.util.List list)` Sets the vm list. |

- - ### Method Detail


    - #### setCloudletList

      ```
      void setCloudletList(java.util.List list)
      ```

      Sets the job list.


    - #### setVmList

      ```
      void setVmList(java.util.List list)
      ```

      Sets the vm list.


    - #### getCloudletList

      ```
      java.util.List getCloudletList()
      ```

      Gets the job list.


    - #### getVmList

      ```
      java.util.List getVmList()
      ```

      Gets the vm list.


    - #### run

      ```
      void run()
               throws java.lang.Exception
      ```

      the main function.

      Throws:
      :   `java.lang.Exception`


    - #### getScheduledList

      ```
      java.util.List getScheduledList()
      ```

      Gets the scheduled jobs.


- Overview
- Package
- Class
- Use
- Tree
- Deprecated
- Index
- Help

- Prev Class
- Next Class

- Frames
- No Frames

- All Classes

- Summary:
- Nested |
- Field |
- Constr |
- Method

- Detail:
- Field |
- Constr |
- Method
